# Genome Analysis of a Novel Broad Host Range Proteobacteria Phage Isolated from a Bioreactor Treating Industrial Wastewater

**DOI:** 10.3390/genes8010040

**Published:** 2017-01-18

**Authors:** Marina de Leeuw, Maayan Baron, Asher Brenner, Ariel Kushmaro

**Affiliations:** 1 Avram and Stella Goldstein-Goren Department of Biotechnology Engineering, Ben-Gurion University of the Negev, P.O. Box 653, Be’er Sheva 8410501, Israel; deleeuw@post.bgu.ac.il (M.d.L.); becherm@post.bgu.ac.il (M.B.); 2 Unit of Environmental Engineering, Faculty of Engineering Sciences, Ben-Gurion University of the Negev, Be’er Sheva 8410501, Israel; brenner@bgu.ac.il; 3 The National Institute for Biotechnology in the Negev, Ben-Gurion University of the Negev, Be’er Sheva 8410501, Israel; 4 The Ilse Katz Center for Meso and Nanoscale Science and Technology, Ben-Gurion University of the Negev, Be’er Sheva 8410501, Israel

**Keywords:** bacteriophage, *Aquamicrobium*, polyvalent, genome, wastewater

## Abstract

Bacteriophages are viruses that infect bacteria, and consequently they have a major impact on the development of a microbial population. In this study, the genome of a novel broad host range bacteriophage, *Aquamicrobium* phage P14, isolated from a wastewater treatment plant, was analyzed. The *Aquamicrobium* phage P14 was found to infect members of different Proteobacteria classes (Alphaproteobacteria and Betaproteobacteria). This phage contains a 40,551 bp long genome and 60% of its genes had blastx hits. Furthermore, the bacteriophage was found to share more than 50% of its genes with several podoviruses and has the same gene order as other polyvalent bacteriophages. The results obtained in this study led to the conclusion that indeed general features of the genome of the *Aquamicrobium* phage P14 are shared with other broad host range bacteriophages, however further analysis of the genome is needed in order to identify the specific mechanisms which enable the bacteriophage to infect both Alphaproteobacteria and Betaproteobacteria.

## 1. Introduction

Bacteriophages (or phages) are considered more abundant and more diverse than bacteria [1]. They are responsible for the majority of bacteria mortality and lysis in aquatic systems, and may contribute to bacterial diversity and biofilm structure [2]. Interest in the presence of phages in wastewater treatment plants has been growing since the 1980s. Hantula et al. and Ewert and Paynter showed that phages are present in activated sludge bioreactors [3,4]. Their work was followed by a few others showing that, over long periods of time, other bioreactors also contained phage populations [5,6,7]. Indeed, the presence of phages in wastewater raises important questions about their influence on the microbial population [8,9] and on the wastewater treatment process.

Reasons for this interest stem from the potential of the phage population to improve the water purification process, based on their capacity to affect the bacterial community [8,9]. For example, they can be used to reduce excess biological sludge [10,11] and excess foaming [12,13] by decreasing the number of foam forming bacteria. Furthermore, phages were suggested to improve bioreactor operation by degrading the bacterially produced exopolysaccharide responsible for biofilm formation [14,15,16]. Nelson et al. and Lu and Collins used phage enzymes to break these exopolysaccharides [14,16]. In addition, Goldman et al. demonstrated that phages isolated from raw sewage samples can inhibit the formation of biofilm on ultra-filtration membranes for several days [17].

In addition, phages were suggested as a method to control different types of bacteria involved in the treatment process, as well as to control pathogen populations in treated water [11]. They can also be used as bioindicators for the presence of pathogens [18]. The latter application can be extremely important since the presence of pathogens may limit the usage of effluent water in agriculture. Experiments aiming at decreasing pathogen populations in food have been performed, with encouraging results [19,20,21]. These results demonstrate how phages could be used for engineering purposes; however, there is need for more knowledge on the phage–bacteria relationship in wastewater treatment facilities.

Phages do not pursue their prey but rather stumble across it. Then, the adsorption of the phage to a bacterium takes place through a receptor on the phage capsid or tail, which attaches to a receptor on the bacterial outer surface. The receptors on the target cell could belong to a variety of families including proteins, carbohydrates and lipids [22]. Thereafter, the phage injects its genetic material into the host bacterial cell and a series of processes depending on the specifics of the infection pathway may lead to immediate replication of the phage (lytic or chronic pathways) or to a lysogenic phase [23,24].

Their attachment mechanisms as well as their dependence on the bacterial host for the replication process, make phages rather specific predators in comparison to other predators in wastewater treatment plants such as protists and predatory bacteria [2]. However, some phages are able to attach to receptors on the outer surface of several bacterial strains. If the phages are also able to penetrate them and use them for replication, these phages are called polyvalent [25].

In this study, we present the complete genome of a polyvalent phage isolated from an industrial wastewater treatment plant [8]. The phage’s capability to infect bacteria from different classes makes it of special interest and the similarities and differences between the phage and other known phages may help gain more knowledge on phage infection abilities.

## 2. Materials and Methods

### 2.1. Sensitivity Test for Several Bacterial Strains

The bacteriophage and eighteen bacterial strains were previously isolated from a full-scale membrane bioreactor treating industrial wastewater at the Neot Hovav industrial park [8]. The bacterial strains were identified by their 16S rRNA sequence (a phylogenetic tree of the different bacterial strains is given in Figure S1) and the phage was isolated from a sample taken 14 days after the bacteria and enriched using *Aquamicrobium* H14 as host [8].

In this study, we initially incubated the different bacterial strains for 48 h in Luria–Bertani (LB) broth (30 °C, constant shaking). Then, 50 μL of the medium containing each strain of bacteria (≈10^7^ colony-forming units (CFU)/mL) were added to 4.5 mL soft LB agar (0.7% agar in LB broth, 45 °C) and plated on 18 LB agar plates. Afterwards, a 7-μL drop containing the phage (≈10^8^ plaque-forming units (PFU)/mL) from a frozen stock (−80 °C) was added to each of the plates. The plates were incubated for 48 h (30 °C) and inhibition of bacterial growth was documented. Out of the five bacterial strains that were found sensitive to the phage, three were incubated in batch conditions together with *Aquamicrobium* phage P14 in order to demonstrate successful phage replication. The concentration of *Aquamicrobium* phage P14 when incubated with *Aquamicrobium* H14 in 100 mL LB broth (30 °C, constant shaking) was shown to increase by more than two orders of magnitude within 24 h (initial bacteria concentration: 3.1 × 10^6^ CFU/mL, initial phage concentration: 5.2 × 10^5^ PFU/mL and final average phage concentration: 1.2 × 10^8^ PFU/mL). Furthermore, when the PFUs of *Aquamicrobium* phage P14 were enumerated after incubation in 10 mL LB broth with *Alcaligenaceae* H5 and *Aquamicrobium* H8 (6 days, 30 °C and constant shaking), the PFU values of *Aquamicrobium* phage P14 were shown to increase by at least one order of magnitude.

### 2.2. Transmission Electron Microscopy

The *Aquamicrobium* phage P14 from a frozen sample was incubated for 48 h (30 °C, constant shaking) with the bacteria *Aquamicrobium* H14 in LB broth. Then, the medium was filtered through a 0.22 µm syringe filter (Durapore^®^ PVDF membrane, Merck Millipore, Billerica, MA, USA). The bacteriophage suspension was fixated using Karnovsky fixative [26], washed twice in cacodylate buffer (pH 7.2, 0.1 M), incubated with osmium tetroxide 1% and washed again. Afterwards, it was dehydrated with ethanol and then incubated with propylene oxide followed by incubation with an araldite mixture. The sample was then incubated at 60 °C for 24 h and placed on copper grids. The grids were negatively stained with phosphotungstic acid and examined by a transmission electron microscope (Tecnai G^2^ 12 TWIN by FEI, Hillsboro, OR, USA).

### 2.3. DNA Purification and Sequencing

In order to enrich the phage sample, a frozen sample of the *Aquamicrobium* phage P14 was thawed and incubated for 48 h (30 °C, constant shaking) with the bacteria *Aquamicrobium* H14 in LB broth. Then, 1.5 mL of the medium were filtered through a 0.22 µm syringe filter (Durapore^®^ PVDF membrane, Merck Millipore). The biomass was further concentrated using Amicon^®^ Ultra-0.5 3K Centrifugal Filters (Merck KGaA, Darmstadt, Germany) yielding 150 µL of concentrate.

The DNA from the concentrate was extracted using the UltraClean Microbial DNA Isolation Kit (MO BIO, Carlsbad, CA, USA) according to manufacturer’s instructions with two modifications: the first step where bacterial cells are pelleted and concentrated was skipped and the elution step was done using only 25 µL of the elution buffer. The DNA concentrations were determined by a NanoDrop 1000 spectrophotometer (Thermo Fisher Scientific, Waltham, MA, USA) and the purified DNA was sequenced using Illumina MiSeq (San Diego, CA, USA), with 250-bp paired-end reads.

### 2.4. Genome Assembly and Analysis

De novo assembly of the phage genome was performed using CLC Genomics Workbench 7.5.1. The paired reads were initially trimmed (the quality limit was set to 0.05 and 600 sequences shorter than 50 nucleotides were discarded). Then, the default settings were used (map reads back to contigs, automatic word size and bubble size, mismatch cost: 2, insertion cost: 3 and deletion cost: 3) with the exceptions of not allowing scaffolding and requiring a length fraction of 0.9 and a similarity fraction of 0.95 for the mapped reads. A total of 3207 reads were mapped to the phage genome (485 single reads and the rest paired reads) and the average coverage for the sequence is 19.56. The sequence of the *Aquamicrobium* phage P14 genome was deposited in the NCBI GenBank database [27], accession: KX660669.

The reading frames were located using Glimmer 3.0 [28]. The genes were then identified using NCBI blastx [29] and searching the non-redundant protein sequences database. For the search the default settings were used (BLOSUM62 matrix, gap costs for existence: 11 and extension: 1, and conditional compositional score matrix adjustment). We used the Expect value (E) as a significance threshold and only results with an E-value smaller than 0.00001 were noted (sequence identity levels ranged between 25% and 69%). If the first hit belonged to a bacterial protein, and there was also a hit belonging to a phage originated protein, both were noted. In addition, tRNAscan-SE [30,31] and BDGP prokaryotic promoter prediction program [32] were used to scan the phage genome for tRNA and promoters.

For multiple sequence alignment, Clustal Omega [33,34] was used. Then, jmodeltest 2.1.10 [35] was used in order to select the best model for construction of a phylogenetic tree by the Bayesian information criterion (BIC) and the Akaike information criterion (AIC). Mega6 [36] was used for the construction of the phylogenetic trees using the selected model.

Moreover, a GC skew analysis and an alignment of different phage genomes was performed using the CGView Server [37]. The blast program used for this alignment was tblastx (translated DNA vs. translated DNA) [29,38]. Finally, we used CoreGenes3.0 [39] for pairwise comparisons with the default “75” score stringency. For the CoreGenes3.0, a file containing the protein sequences encoded by the viral genome was created using ExPASy (SIB Swiss Institute of Bioinformatics) [40].

## 3. Results and Discussion

The polyvalent *Aquamicrobium* phage P14 was found, using the spot test, to infect two different *Aquamicrobium* strains named H8 (GQ254278) and H14 (GQ254284), and three different *Alcaligenaceae* strains named H5 (GQ254275), H13 (GQ254283) and H17 (GQ254287). The phage was shown to grow in LB broth with the hosts: *Aquamicrobium* H8, *Aquamicrobium* H14 and *Alcaligenaceae* H5. The *Aquamicrobium* genus belongs to the Alphaproteobacteria class while the *Alcaligenaceae* family belongs to the Betaproteobacteria class. The phage’s ability to infect bacteria from different classes makes it of special interest. This is due to its possible influence on the wastewater treatment process from which it was isolated, but also since its broad host range may help understand the infection mechanisms involved.

**Figure 1:**
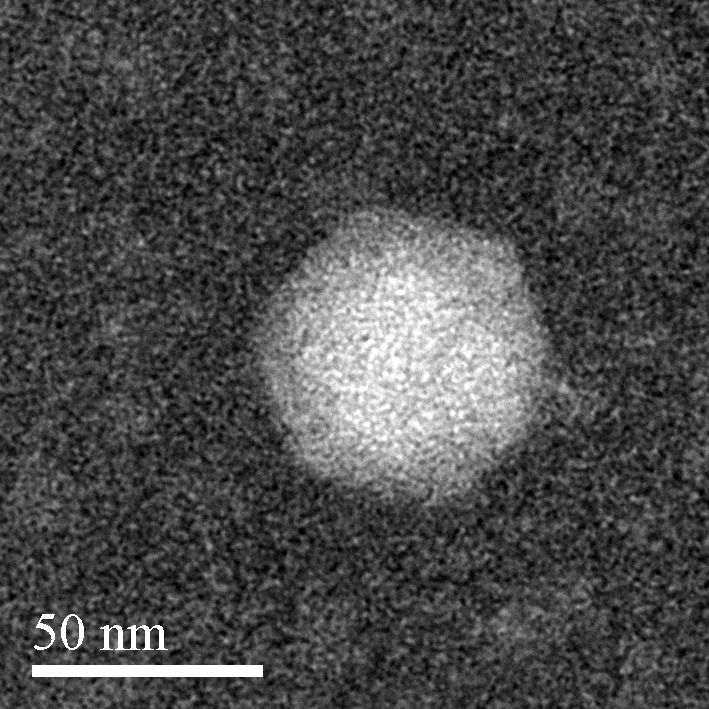
Negatively stained transmission electron microscopy image of the *Aquamicrobium* phage P14.

The *Aquamicrobium* phage P14 has an icosahedral phage head with a diameter of approximately 50 nm, as seen in Figure 1. In addition, a possible short tail could be spotted to the right of the head. This correlates with the analysis of the phage genome that strongly suggests that the phage belongs to the *Podoviridae* family [41].

### 3.1. General Features of the Phage Genome

**Figure 2:**
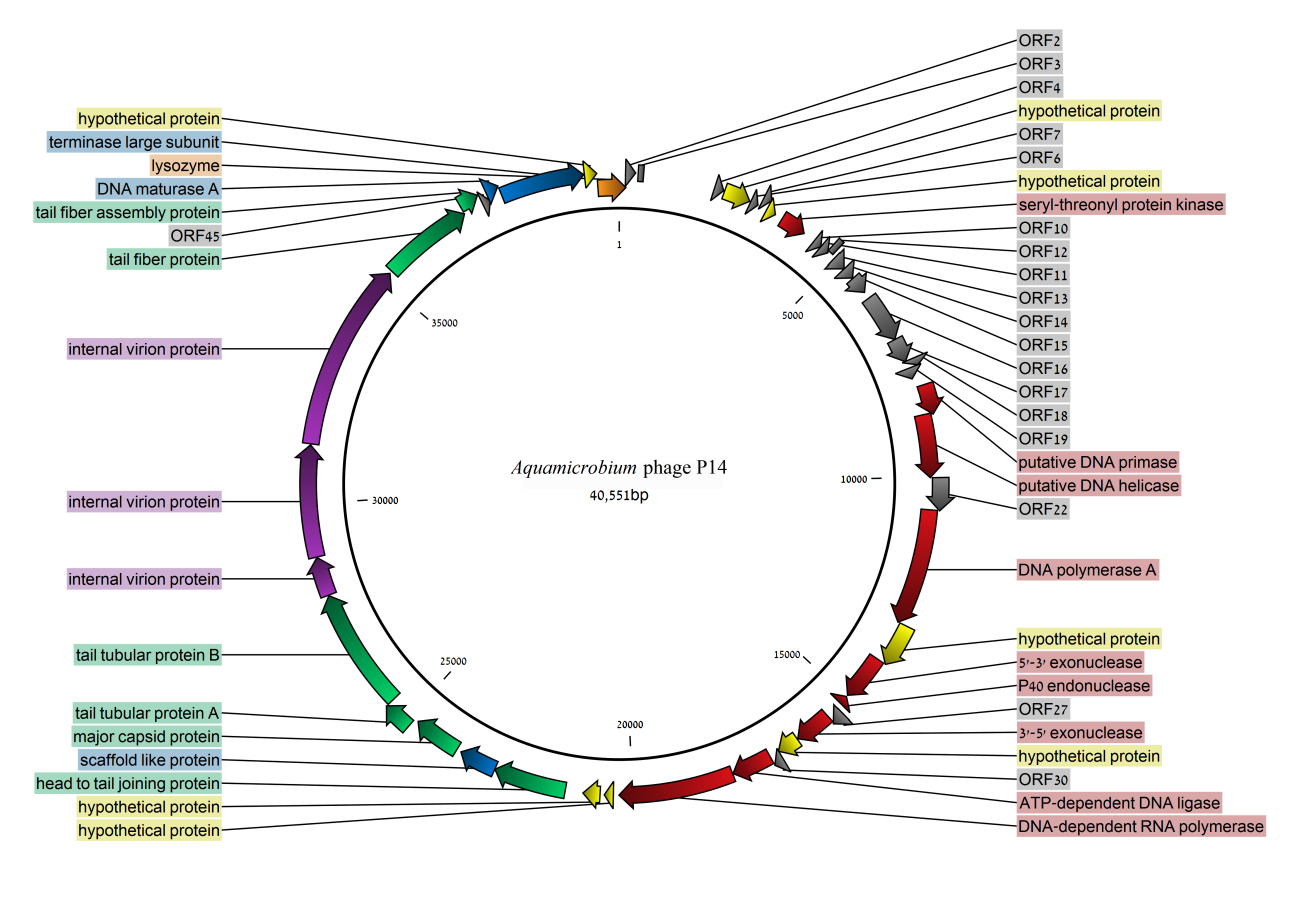
The genome of the *Aquamicrobium* phage P14. Open reading frames (ORFs) with blastx hits [29] are indicated in color. **Yellow**: hypothetical proteins; **orange**: lysozyme; **blue**: phage packaging (assembly); **purple**: internal virion proteins; **green**: structure proteins; and **red**: all other proteins.

The genome of the *Aquamicrobium* phage P14 was found to be 40,551 bp long and its GC content is 57.8% (the GC content and skew are shown in Figure 6). No tRNA coding regions were found. Forty-eight open reading frames (ORFs) were listed in the final predicted genes file using the default settings. Out of the 48 ORFs 29 (60%) were identified (Figure 2) searching the non-redundant database using blastx [29]. As can be seen in Table 1, all of the genes were closely similar to genes found in other phages (the search was not restricted to virus databases at any point). Only in six cases, there was a protein with a bacterial origin with a higher E-value than phage originated proteins (Table 1). Additionally, in 17 ORFs, domains were identified (Table S1).

**Figure 3:**
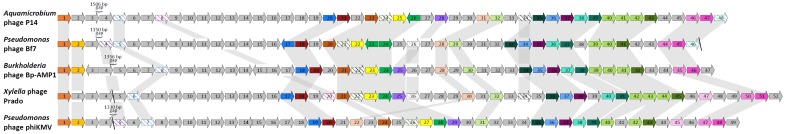
Genomic organization of the *Aquamicrobium* phage P14 compared to the *Pseudomonas* phage Bf7 (NC_016764.1), the *Burkholderia* phage Bp-AMP1 (HG793132.1), *Xylella* phage Prado (NC_022987.1) and *Pseudomonas* phage phiKMV (NC_005045.1). Coding sequences encoding proteins with similar functions are colored in the same color. Hypothetical proteins are marked by diagonal lines. A match between the proteins was either defined by similarity of the amino acid sequences (blastx hits with E-value < 0.00001) or proteins with the same function as defined in their entry.

A relatively large gap of 1506 bp was found between ORF3 and ORF4 with no coding sequences. This gap does not contain any open reading frames and has no blast and blastx matches. However, such a non-protein coding section is present in other phages roughly in the same location (Figure 3). Examples are the genome of the *Pseudomonas* phage phiKMV (NC_005045.1) where the gap is 1330 bp long, the genome of *Pseudomonas* phage Bf7 (NC_016764.1) where the gap is 1150 bp long, and the genome of the *Burkholderia* phage Bp-AMP1 (HG793132.1) where the gap is 1356 bp long. In the case of the *Pseudomonas* phage phiKMV, four promoters were found to be present in this DNA section [42] which is located near the 5′-end of the linear genome. Using the BDGP prokaryotic promoter prediction program [32] we found five promoters between ORF3 and ORF4 of *Aquamicrobium* phage P14, suggesting this region has regulatory characteristics.

### 3.2. Coding Sequences Organization

The coding sequences can be divided into three major groups with few exceptions. The early class contains the DNA helicase (ORF20), DNA primase (ORF21) and the DNA-dependent RNA polymerase (ORF32) [43]. The DNA-dependent RNA polymerase is not located next to the DNA helicase and DNA primase but is located further away after the middle class genes. This is also the case in phiKMV-like phages such as the broad host range LIMElight phage of *Pantoea agglomerans* [44] and the PPA-ABTNL phage which was found to infect 14 strains of *Pseudomonas aeruginosa* [43].

The second gene cluster is responsible for DNA replication and repair and is called the middle class. It includes a DNA polymerase, an exonuclease 5′–3′, an endonuclease, an exonuclease 3′–5′ and an ATP-dependent DNA ligase. The third gene cluster is responsible for the late phase genes encoding proteins which are responsible for the viral assembly and encoding structure proteins.

As can be seen in Figure 3, the general coding sequences order in the genome of the *Aquamicrobium* phage P14 is similar to the gene organization of several phages. These include the *Pseudomonas* phage phiKMV and other podoviruses classified as phiKMV-like. A change in the location of the gene encoding the ATP dependent DNA ligase, located in ORF31 of *Aquamicrobium* phage P14, can be observed in Figure 3. The gene has the same location as it has in the genomes of the other phiKMV-like phages presented, however, in the case of the *Pseudomonas* phage phiKMV, the gene encoding this protein is located at the beginning of the middle class.

Not only the order of recognized proteins is preserved between the different phages, the location of several hypothetical proteins is preserved as well. This indicates their function is probably important although unknown. In only one case a coding sequence of a hypothetical protein changed its location in comparison to the phages shown in Figure 3. This is the coding sequence of a hypothetical protein located in ORF8 of *Aquamicrobium* phage P14. This gene was also found in the genome of the *Xylella* (Gammaproteobacteria) phage Prado in ORF20, between the early class gene cluster and the middle class gene cluster.

In the case of all five phages presented in Figure 3, there is a group of hypothetical proteins located in the left of the figure with an unknown function. It is nowadays assumed that the compactness of phages does not allow them to carry unnecessary genetic material. Therefore, this group should be further analyzed and identified.

### 3.3. Early Class

The coding sequences of proteins belonging to the early class are located in the phage genome in ORF20, ORF21 and ORF32. These proteins are the DNA primase, DNA helicase and a DNA-dependent RNA polymerase. The binding of DNA primase to DNA helicase enables the synthesis of RNA primers as an early step for DNA replication. The DNA primase shares 48% identity (95% query coverage) with the DNA primase of the *Burkholderia* (Betaproteobacteria) phage Bp-AMP4, a podovirus with double-stranded DNA (dsDNA) and the DNA helicase shares 51% identity (99% query coverage) with the DNA helicase of *Caulobacter* (Alphaproteobacteria) phage Cd1, a podovirus which infects *Caulobacter crescentus*.

The DNA dependent RNA polymerase produces RNA using the DNA of the genome as a template. The coding sequence of the RNA polymerase is located in ORF32 and the translated protein sequence was found to be highly similar, 60% (99% query coverage), to the RNA polymerase of *Caulobacter* phage Percy, a podovirus which infects the Gram-negative bacteria *Caulobacter crescentus* [45].

### 3.4. DNA Replication and Repair (Middle Genomic Region)

DNA replication and repair genes include DNA polymerase, exonucleases, endonuclease and DNA ligase. The DNA polymerase found to be encoded by ORF23 has 57% identity (query coverage of 99%) to the DNA polymerase of the *Ralstonia* phage RSJ2, a lytic podovirus, which infects several Thai and Japanese strains of *Ralstonia solanacearum* [46].

The exonuclease in ORF25 was found most similar (47% identity, 97% query coverage) to the exonuclease of *Burkholderia* phage Bp-AMP1. It is also very similar to the 5′–3′ exonuclease of *Xylella* phage Paz (45% identity, 92% query coverage). This enzyme is responsible for the cleavage of RNA primers upstream of the DNA polymerase.

Another exonuclease was found in ORF28 and shares 64% identity (99% query coverage) with an RNase H superfamily protein of the Gram-negative bacteria *Burkholderia cepacia*. This protein has a 3′–5′ exonuclease domain (Table S1). Interestingly, the exonuclease was also found to be similar to exonucleases of phages infecting members of the *Burkholderia* genus. It shares a 57% identity (93% query coverage) with the exonuclease of *Burkholderia* phage JG068, a lytic podovirus with a broad host range including: *Burkholderia multivorans*, *Burkholderia cenocepacia*, *Burkholderia stabilis* and *Burkholderia dolosa* [47].

An endonuclease found to be encoded by ORF26 was found to be similar to the endonuclease of *Xanthomonas* (Gammaproteobacteria) phage phiL7 with a 61% query identity (100% query coverage). The *Xanthomonas* phage phiL7 is a lytic phage, which has a long tail and belongs to the *Siphoviridae* family. It infects the plant pathogen *Xanthomonas campestris* [48].

DNA ligase is known to be present in dsDNA phages such as the Enterobacteria phage T4 [49] and is capable of repairing single strand breaks in dsDNA. The DNA ligase of our *Aquamicrobium* phage P14 is 45% identical to the DNA ligase of the *Xylella* phage Prado (98% query coverage), a broad host range podovirus known to infect members of the *Xylella* genus and the *Xanthomonas* genus [50].

### 3.5. Packaging Related Genes (Late Genomic Region)

During the replication of the phage its dsDNA has to be packed. This process involves the terminase proteins, which are ATP driven and are responsible for slicing the dsDNA into the final genome sized sequences, which are then incorporated into an empty capsid. The terminase large subunit (DNA maturase B) was found to be encoded by ORF47, and is highly similar to the terminase large subunit of *Caulobacter* phage Percy (57% identity, 99% query coverage). A putative DNA maturase A is encoded by ORF46. However, the closest identity, which was found to the DNA maturase A of *Caulobacter* phage Cd1, is only 41% with 94% query coverage. Another packaging related gene is the scaffold protein in ORF36. This protein is crucial for the formation of the viral procapsid [51]. It was found to share 48% identity (45% query coverage) with the scaffold protein of *Caulobacter* phage Cd1, as well.

The fact that all the packaging related proteins were found to be most similar to those of *Caulobacter* phages may indicate that the whole region is highly conserved which might be a result of lateral gene transfer [52]. However, it is possible that this result was obtained due to lack of sequences in the database, and that in the future these proteins will be found most similar to proteins of distinct phages.

### 3.6. Internal Virion Genes

Three internal virion genes were located in the phage genome (ORF40, ORF41 and ORF42). ORF40 and ORF41 have a rather low identity of 25% to an internal virion protein of *Xylella* phage Prado (99% query coverage) and *Xylella* phage Paz (88% query coverage), respectively. The longest internal virion gene is located in ORF 42 and the encoded protein has 41% identity with an internal virion protein of *Caulobacter* phage Cd1 (99% query coverage).

### 3.7. Phage Capsid and Tail Genes

Six genes related to the phage capsid and tail were identified in the phage genome and are located in two distinct regions. The first region includes the major capsid protein, the head to tail connector, the tail fiber protein and the tail fiber assembly protein. The major capsid protein shares 50% identity with the capsid protein of the *Caulobacter* phage Cd1. The head tail connector (ORF 35) is similar to the one found in the genome of the *Caulobacter* phage Percy, with a 55% similarity. The tail tubular proteins A (ORF38) and B (ORF39) share 43% identity and 44% identity to the genes in the *Burkholderia* phage Bp-AMP1 and *Caulobacter* phage Cd1, respectively.

**Figure 4:**
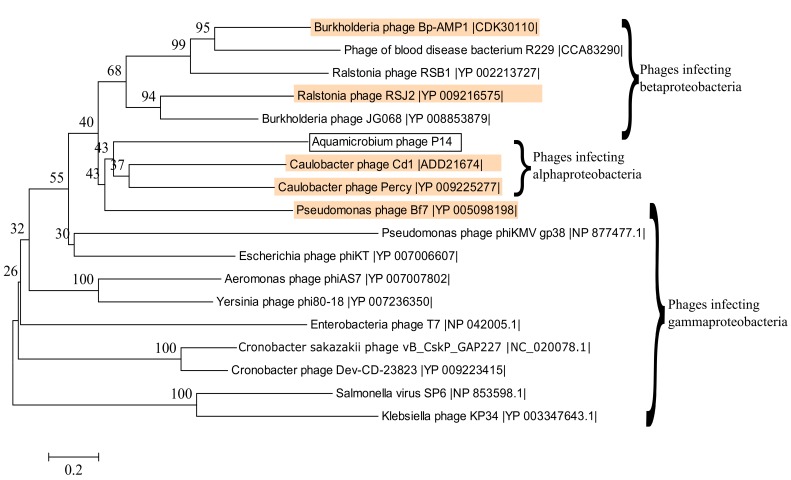
Phylogenetic tree constructed based on the DNA sequences encoding the tail fiber proteins of several phages. Phages that are also present in Table 2 are highlighted in orange. The best model for the phylogenetic tree construction was found by jmodeltest 2.1.10 [35] to be GTR+G+I. The model was then constructed using Mega6 [36] with 500 bootstrap replications.

The second region includes the tail fiber protein (ORF43) and the tail fiber assembly protein (ORF44). These proteins were found similar to those in the *Caulobacter* phage Cd1 (38% identity, 39% query coverage) and the Mediterranean phage uvMED (42% identity, 94% query coverage), respectively. A phylogenetic tree constructed for the DNA sequence encoding the tail fiber protein is shown in Figure 4. As can be seen, the DNA sequence encoding the tail fiber protein is related to the DNA sequence encoding the tail fiber protein of phages infecting Alphaproteobacteria, Betaproteobacteria and Gammaproteobacteria. Interestingly the majority of the blastx hits were at the N-terminus of the tail fiber protein. Furthermore, there was a match (E-value: 1.28 × 10^−11^) to the phage T7 tail fiber protein superfamily (pfam03906) at the N-terminus. This is where the tail fiber protein of the bacteriophage T7 attaches to the phage’s tail [53]. Only one phage had sequence similarities, although the query coverage was rather low, at the C-terminus: a putative phage tail protein found in the genome of the bacteria *Selenomonas ruminantium* (42% identity, 18% query coverage, E-value: 7 × 10^−16^, accession no.: WP_014425996.1).

In the case of the phage capsid and tail genes, again, almost all of the proteins were found to be most similar to those of *Caulobacter* phages [45]. The tail tubular protein A was found to be most similar to that of *Burkholderia* phage Bp-AMP1 but is actually also very similar to the protein of *Caulobacter* phage Percy (93% query coverage, 44% identity). This may indicate that the gene cluster is highly conserved which might be a result of lateral gene transfer of the whole section [52]. However, the limitations of the database should also be taken into account as explained earlier.

### 3.8. Lysozyme

The lysozyme is an enzyme which is capable of damaging the bacterial wall. Therefore, it has a major impact on the ability of the phage DNA to penetrate the bacterial cell, release the new virions and degrade biofilms produced by the bacteria [14]. In the case of the *Aquamicrobium* phage P14, its lysozyme sequence was found to have 43% identity (79% query coverage) with the lysozyme of the rod shaped Gram-negative bacteria *Serratia marcescens*. It was also found to be highly similar to lysozyme sequences identified in other phages such as the polyvalent *Pseudomonas* phage Bf7 (35% identity, 79% query coverage), which infects several members of *Pseudomonas* genus [54].

### 3.9. Seryl/threonyl Protein Kinase

Seryl/threonyl protein kinase is a protein that phosphorylates serine and threonine on target proteins. A putative seryl/threonyl protein kinase is possibly encoded in ORF9 where the sequence was found similar (29% identity, 72% query coverage) to the seryl/threonyl protein kinase of *Erwinia* phage FE44.

### 3.10. Alignment to Other Phages

**Figure 5:**
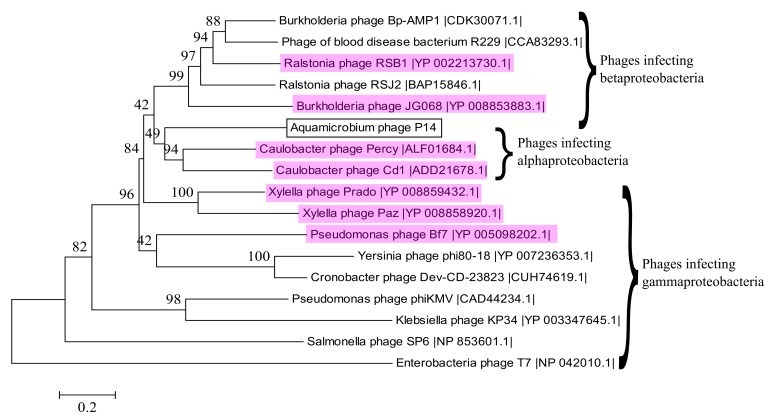
Phylogenetic relationship between selected terminase large subunit DNA sequences. Phages that are classified as phiKMV-like are highlighted in magenta. The best model for the phylogenetic tree construction was found by jmodeltest 2.1.10 [35] to be GTR + G + I. The model was then constructed using Mega6 [36] with 500 bootstrap replications.

We used CoreGenes3.0 for pairwise aligning of our phage to several phage genomes from Table 1. As can be seen in Table 2, the highest gene correlation was found when our phage was compared to other phiKMV-like phages. In addition, the terminase large subunit was used to construct a phylogenetic tree as shown by Serwer et al. [55] and Fouts et al. [56]. The phylogenetic tree shows that the DNA sequence of the terminase large subunit is similar to the same sequence of several phiKMV-like viruses (Figure 5). Out of the sixteen phages shown, fifteen are classified as belonging to the *Podoviridae* family, from which seven are classified as phiKMV-like phages (highlighted in magenta). The remaining phage, a blood disease bacterium R229 phage [57], is not classified at all.

In addition, three different phages from Table 2, infecting different hosts, but sharing several genes with the *Aquamicrobium* phage P14 were aligned using the CGView Server [37]: (1) The *Pseudomonas* phage Bf7 is a lytic phage belonging to the *Podoviridae* family with a dsDNA genome of 40,058 bp [54]. It was found to infect 16 strains of *Pseudomonas*. (2) The *Xylella* phage Prado, which also has a broad host range, infects *Xylella fastidiosa* as well as members of the *Xanthomonas* spp. It is a lytic podovirus with a genome of 43,940 bp (63.0% GC content). (3) The *Burkholderia* phage Bp-AMP1 is a podovirus that was found to infect 11 strains of *Burkholderia pseudomallei* [58]. This phage has a 42,409 bp long genome (61.75% GC content) and was found to have a temperature-dependent infection cycle [59].

**Figure 6:**
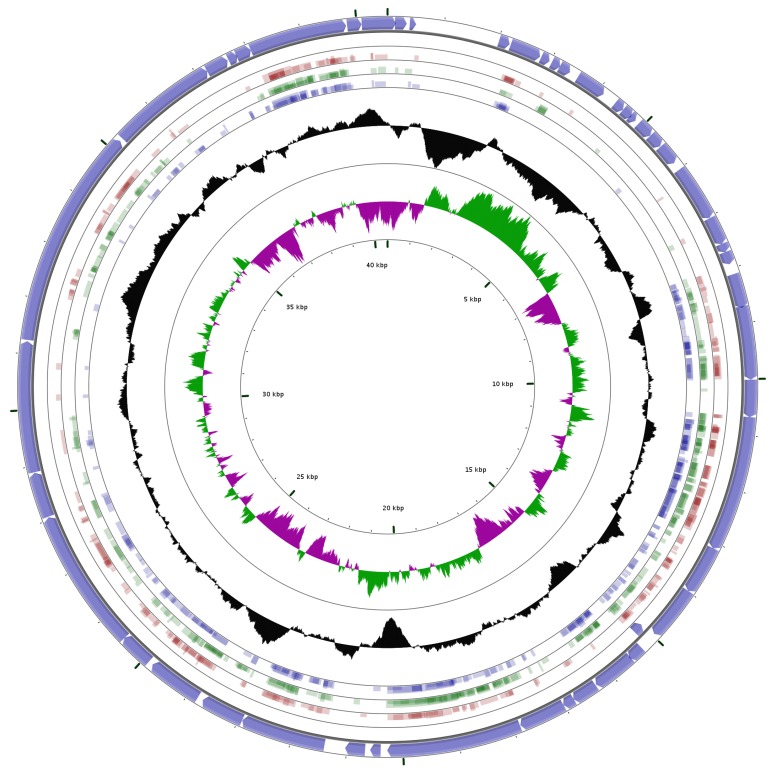
Alignment using tblastx (minimum E-value: 0.00001, minimum aligned query length: 10 amino acids, and minimum identity cutoff: 25%) of the *Aquamicrobium* phage P14 genome against the genomes of *Pseudomonas* phage Bf7 (**red**; NC_016764.1); *Xylella* phage Prado (**green**; NC_022987.1) and the *Burkholderia* phage Bp-AMP1 (**blue**; HG793132.1); The outer circle shows the coding sequences marked by arrows. The two inner circles show the GC content (**black**); and GC skew + (**green**) and the GC skew − (**magenta**).

As can be seen in Figure 6, when the genomes of the three phages were aligned to the genome of *Aquamicrobium* phage P14, matches were found for most of the coding sequences. All the areas without matches to these three phages are areas coding for hypothetical proteins without a known function. We believe that the similarities and differences should be further analyzed in order to identify the characteristics which make these phages polyvalent. Moreover, for this alignment, tblastx was used, which means that differences in the nucleic acid sequence that do not influence the amino acid sequence are practically ignored.

## 4. Conclusions

*Aquamicrobium* phage P14 is capable of infecting bacteria from different classes. Sixty percent of the phage’s genes were identified using blastx and were found to have similarities to other phages. Classification of the phage by its transmission electron microscopy image led to the conclusion that the phage is a podovirus. Furthermore, the phylogenetic tree by the terminase large subunit and the analysis of the genes suggest the phage should be classified as phiKMV-like. In addition, the phage shares more than 45% of its genes with other polyvalent phages and has a conserved gene order similar to phiKMV-like phages with a broad host range. Further analysis of the similarities and differences between the *Aquamicrobium* phage P14 and other polyvalent phages could supply answers regarding the mechanisms underlying its infection pathways. Such research may have implications in the fields of wastewater treatment, where polyvalent phages were suggested as tools for improving the process, agriculture and medicine.

## Figures and Tables

**Table 1:** Open reading frames on the genome of the *Aquamicrobium* phage P14 and their blastx [29] hits. Sources of similar proteins that are phages are marked in bold. Only blastx matches with an E-value smaller than 0.00001 are listed.

ORF	Start	End	Strand	Protein Length	Blastx Match	E-Value	Source	Accession No.
ORF1	40092	149	+	202	lysozyme	1.00 × 10^−24^	*Serratia marcescens*	WP_043138231.1
					putative lysozyme	4.00 × 10^−22^	***Pseudomonas* phage Bf7**	YP_005098158.1
ORF2	133	342	+	69	none			
ORF3	395	508	+	37	none			
ORF4	2014	2211	+	65	none			
ORF5	2232	2795	+	187	hypothetical protein	2.00 × 10^−39^	***Pseudomonas* phage**	YP009151803.1
ORF6	2818	2982	+	54	none			
ORF7	3027	3191	+	54	none			
ORF8	3188	3394	+	68	hypothetical protein	1.00 × 10^−18^	***Xylella* phage Prado**	YP008859401.1
ORF9	3559	4107	+	182	seryl/threonyl protein kinase	7.00 × 10^−5^	***Erwinia* phage FE44**	YP008766718.1
ORF10	4367	4549	+	60	none			
ORF11	4551	4742	+	63	none			
ORF12	4718	4843	+	41	none			
ORF13	4919	5215	+	98	none			
ORF14	5218	5475	+	85	none			
ORF15	5486	5863	+	125	none			
ORF16	6011	7024	+	337	none			
ORF17	7028	7534	+	168	none			
ORF18	7531	7692	+	53	none			
ORF19	7689	7919	+	76	none			
ORF20	8101	8727	+	208	putative DNA primase	2.00 × 10^−53^	***Burkholderia* phage Bp-AMP4**	CDL65258.1
ORF21	8697	10010	+	437	putative DNA helicase	4.00 × 10^−146^	***Caulobacter* phage Cd1**	ADD21652.1
ORF22	10012	10680	+	222	none			
ORF23	10677	13109	+	810	DNA polymerase A family protein	0.0	*Burkholderia pseudomallei* BDU 2	KGV49475.1 pfam00476
					putative DNA polymerase	0.0	***Ralstonia* phage RSJ2**	BAP15824.1
ORF24	13106	13984	+	292	hypothetical protein	8.00 × 10^−60^	*Burkholderia* sp. 2002721687	AJY44207.1
					hypothetical protein	1.00 × 10^−46^	***Pseudomonas* phage Bf7**	YP005098178.1
ORF25	13984	14958	+	324	putative exonuclease	3.00 × 10^−76^	***Burkholderia* phage Bp-AMP1**	CDK30092.1
ORF26	15181	14993	−	62	DNA endonuclease P40	3.00 × 10^−17^	***Xanthomonas* phage phiL7**	YP002922654.1
ORF27	15292	15573	+	93	none			
ORF28	15552	16331	+	259	RNase H superfamily protein	3.00 × 10^−119^	*Burkholderia cepacia*	WP_060050861.1 pfam13482
					DNA exonuclease	2.00 × 10^−102^	***Burkholderia* phage JG068**	YP008853863.1
ORF29	16345	16809	+	154	hypothetical protein	4.00 × 10^−67^	*Pseudomonas composti*	WP061238191.1
					hypothetical protein	3.00 × 10^−29^	***Mycobacterium* phage Omega**	NP818474.1
ORF30	16806	17015	+	69	none			
ORF31	17015	17860	+	281	ATP-dependent DNA ligase	8.00 × 10^−67^	***Xylella* phage Prado**	YP008859411.1
ORF32	17869	20283	+	804	DNA-dependent RNA polymerase	0.0	***Caulobacter* phage Percy**	ALF01667.1
ORF33	20400	20588	+	62	hypothetical protein	8.00 × 10^−5^	***Caulobacter* phage Percy**	ALF01668.1
ORF34	20677	21033	+	118	hypothetical protein	1.00 × 10^−14^	***Caulobacter* phage Cd1**	ADD21663.1
ORF35	21404	22930	+	508	head-to-tail joining protein	2.00 × 10^−172^	***Caulobacter* phage Percy**	ALF01671.1
ORF36	22934	23722	+	262	hypothetical protein	2.00 × 10^−21^	*Burkholderia thailandensis* MSMB43	EIP87426.1
					scaffold-like protein	7.00 × 10^−20^	***Caulobacter* phage Cd1**	ADD21667.1
ORF37	23820	24815	+	331	major capsid protein	2.00 × 10^−98^	***Caulobacter* phage Cd1**	ADD21668.1
ORF38	24897	25502	+	201	tail tuber protein A	4.00 × 10^−48^	***Burkholderia* phage Bp-AMP1**	CDK30105.1
ORF39	25499	28045	+	848	tail tubular protein B	0.0	***Caulobacter* phage Cd1**	ADD21670.1
ORF40	28057	28875	+	272	hypothetical protein	1.00 × 10^−10^	***Caulobacter* phage Cd1**	ADD21671.1
					internal virion protein	7.00 × 10^−7^	***Xylella* phage Prado**	YP008859423.1
ORF41	28885	31227	+	780	hypothetical protein	2.00 × 10^−43^	***Caulobacter* phage Cd1**	ADD21672.1
					internal virion protein	1.00 × 10^−38^	***Xylella* phage Paz**	YP008858912.1
ORF42	31250	35209	+	1319	internal virion protein	0.0	***Caulobacter* phage Cd1**	ADD21673.1
ORF43	35267	37195	+	642	tail fiber protein	5.00 × 10^−32^	***Caulobacter* phage Cd1**	ADD21674.1
ORF44	37195	37605	+	136	tail fiber assembly protein	3.00 × 10^−24^	**Mediterranean phage uvMED**	BAQ90231.1
ORF45	37625	37795	+	56	none			
ORF46	37782	38057	+	91	putative DNA maturase A	1.00 × 10^−10^	***Caulobacter* phage Cd1**	ADD21677.1
ORF47	38057	39814	+	585	terminase large subunit	0.0	***Caulobacter* phage Percy**	ALF01684.1
ORF48	39831	40088	+	85	hypothetical protein	4.00 × 10^−4^	***Pseudomonas* phage Bf7**	YP005098203.1

**Table 2:** CoreGenes3.0 correlation values for *Aquamicrobium* phage P14 versus several phages from Table 1.

Phage	Hosts	CoreGenes3.0 Correlation	Genome Length	GC Content	Classification
*Aquamicrobium* phage P14	*Aquamicrobium* strains and *Alcaligenaceae* strains	-	40,551 bp	57.8%	*Podoviridae* phiKMV-like phages
*Burkholderia* phage Bp-AMP1 (HG793132.1)	*Burkholderia pseudomallei* strains	47.92%	42,409 bp	61.8%	*Podoviridae*
*Burkholderia* phage Bp-AMP4 (HG796221.1)	*Burkholderia pseudomallei*	47.92%	42,112 bp	61.8%	*Podoviridae*
*Caulobacter* phage CD1 (GU393987.1)	*Caulobacter crescentus*	54.17%	41,581 bp	61.2%	*Podoviridae* phiKMV-like phages
*Caulobacter* phage Percy (NC_029092.1)	*Caulobacter crescentus*	47.27%	44,773 bp	60.9%	*Podoviridae* phiKMV-like phages
*Erwinia* phage FE44 (NC_022744.1)	*Erwinia carotovora*	22.92%	39,860 bp	48.6%	*Podoviridae* T7likevirus
*Pseudomonas* phage Bf7 (NC_016764.1)	*Pseudomonas* strains	47.92%	40,058 bp	58.4%	*Podoviridae* phiKMV-like phages
*Ralstonia* phage RSJ2 (NC_028988.1)	*Ralstonia solanacearum* strains	50.00%	44,360 bp	60.9%	*Podoviridae*
*Xanthomonas* phage phiL7 (NC_012742.1)	*Xantomonas campestris*	20.83%	44,080 bp	55.6%	*Siphoviridae*
*Xylella* phage Paz (NC_022982.1)	*Xylella fastidiosa* and *Xantomonas*	47.92%	43,869 bp	60.2%	*Podoviridae* phiKMV-like phages
*Xylella* phage Prado (NC_022987.1)	*Xylella fastidiosa* and *Xantomonas*	52.08%	43,940 bp	63.0%	*Podoviridae* phiKMV-like phages

## Supplementary Materials

The following are available online at www.mdpi.com/2073-4425/8/1/40/s1, Figure S1: A phylogenetic tree of the bacteria, numbered H1–H18, tested in this work, Table S1: Open reading frames on the genome of the *Aquamicrobium* phage P14 and their domain matches found using blastx.
