# Supplementary material for: Genome Analysis of a Novel Broad Host Range Proteobacteria Phage Isolated from a Bioreactor Treating Industrial Wastewater
Source: Genes (Basel). 2017 Jan 18;8(1):40. doi: 10.3390/genes8010040 (PMC5295034; doi:10.3390/genes8010040)
Supplement: Supplementary file 1 [file genes-08-00040-s001.docx]

Supplementary Materials: Genome Analysis of a Novel Broad Host Range Proteobacteria Phage Isolated from a Bioreactor Treating Industrial Wastewater

**Marina de Leeuw, Maayan Baron, Asher Brenner and Ariel Kushmaro**


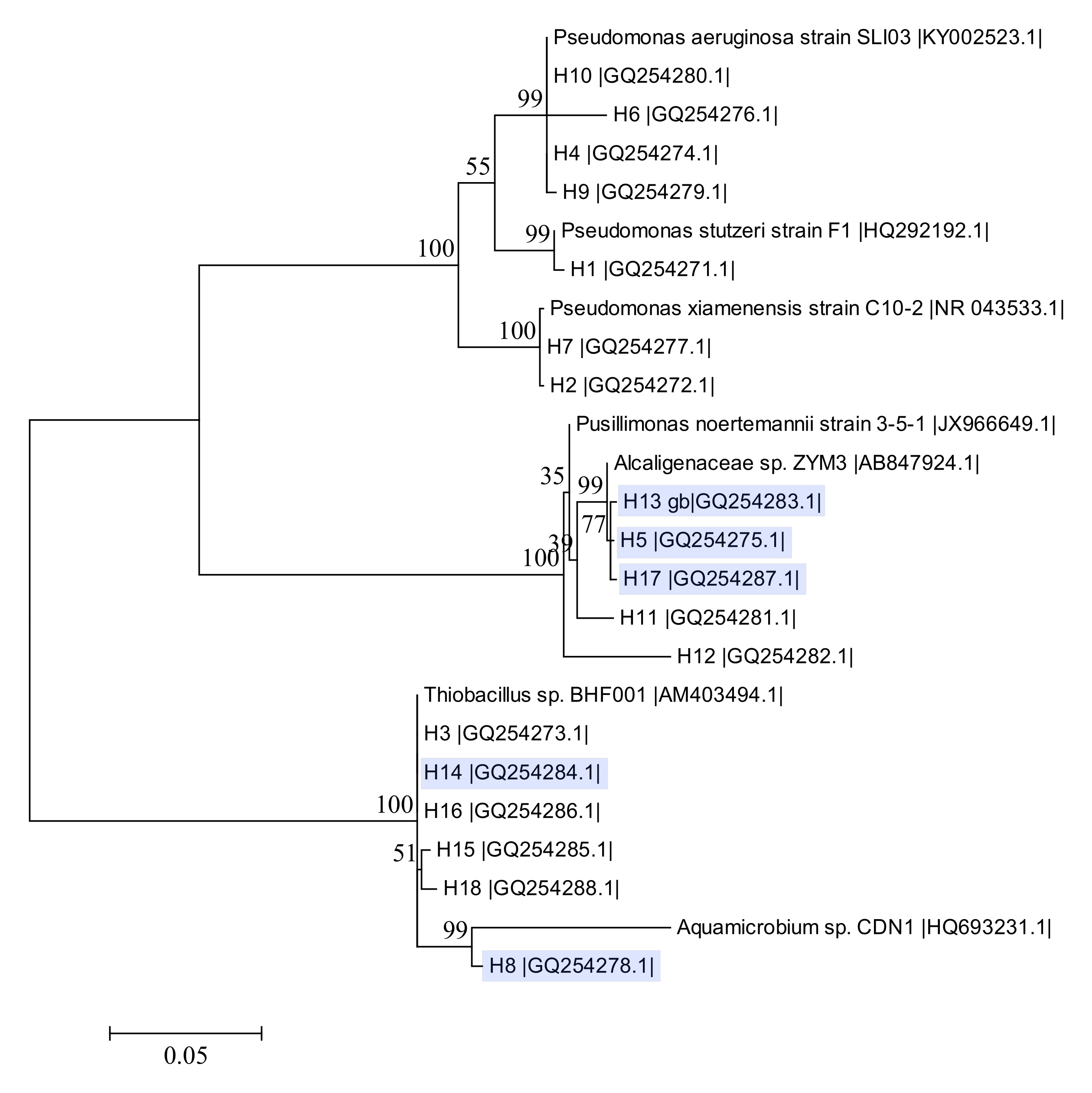


**Figure S1.** A phylogenetic tree of the bacteria, numbered H1–H18, tested in this work. The five bacterial strains that were found sensitive to *Aquamicrobium* phage P14 are highlighted in blue. The 16S rRNA sequences of the different bacterial strains were aligned by Clustal Omega [33,34] using the default settings. The best model for phylogenetic tree construction, TrN + G, was selected by jmodeltest 2.1.10 [35]. It was ranked as the best model by the Bayesian information criterion (BIC) and ranked 5th by the Akaike information criterion (AIC). Finally, the tree was constructed using MEGA6 [36] with 500 bootstrap replications.

**Table S1.** Open reading frames on the genome of the *Aquamicrobium* phage P14 and their domain matches found using blastx [29]. Only domain matches with E-values smaller than 0.0001 are listed.

| **ORF** | **Start** | **End** | **Strand** | **Protein Length** | **Domain Hits** | **E-Value** | **Interval** | **Accession** |
| --- | --- | --- | --- | --- | --- | --- | --- | --- |
| ORF1 | 40092 | 149 | + | 202 | phage lysozyme | 2.96 × 10^-14^ | 52-143 | pfam00959 |
|  |  |  |  |  | RrrD Phage-related lysozyme (muramidase), GH24 family | 6.81 × 10^-42^ | 22–165 | COG3772 |
|  |  |  |  |  | endolysin autolysin | 1.36 × 10^-40^ | 22–159 | cd00737 |
| ORF5 | 2232 | 2795 | + | 187 | PHA01782 superfamily | 3.17 × 10^-49^ | 22–181 | PHA01782 |
| ORF9 | 3559 | 4107 | + | 182 | PHA00451 protein kinase | 2.13 × 10^-10^ | 54–182 | PHA00451 |
| ORF20 | 8101 | 8727 | + | 208 | TOPRIM_primases | 1.18 × 10^-07^ | 109-178 | cd01029 |
|  |  |  |  |  | putative DnaG-like primase | 5.33 × 10^-09^ | 89–195 | PHA02031 |
|  |  |  |  |  | DnaG DNA primase | 1.91 × 10^-08^ | 40–196 | COG0358 |
|  |  |  |  |  | DNA primase | 1.59 × 10^-05^ | 39–187 | TIGR01391 |
| ORF21 | 8697 | 10010 | + | 437 | AAA domain | 5.66 × 10^-07^ | 154–352 | pfam1348 |
|  |  |  |  |  | DnaB helicase C terminal domain | 2.76 × 10^-06^ | 171–375 | cd00984 |
|  |  |  |  |  | DnaB Replicative DNA helicase | 2.29 × 10^-13^ | 118–389 | COG0305 |
|  |  |  |  |  | phage replicative helicase, DnaB family, HK022 subfamily | 4.48 × 10^-08^ | 93–389 | TIGR03600 |
|  |  |  |  |  | replicative DNA helicase; Provisional | 5.24 × 10^-06^ | 125–389 | PRK07004 |
| ORF23 | 10677 | 13109 | + | 810 | DNA polymerase family A | 1.35 × 10^-30^ | 386–762 | pfam00476 |
|  |  |  |  |  | DNA polymerase A Aquificae like | 2.76 × 10^-25^ | 378–771 | cd08639 |
|  |  |  |  |  | DNA polymerase A domain | 1.31 × 10^-21^ | 480–730 | smart00482 |
|  |  |  |  |  | DNA polymerase I–3′–5′ exonuclease and polymerase domains | 1.79 × 10^-29^ | 386–774 | COG0749 |
|  |  |  |  |  | DNA polymerase I | 4.53 × 10^-28^ | 388–770 | TIGR00593 |
|  |  |  |  |  | bifunctional 3′–5′ exonuclease/DNA polymerase; Provisional | 1.50 × 10^-27^ | 459–769 | PRK14975 |
| ORF24 | 13106 | 13984 | + | 292 | hypothetical protein | 3.05 × 10^-41^ | 13–288 | PHA02030 |
| ORF25 | 13984 | 14958 | + | 324 | 5′–3′ exonuclease, C-terminal SAM fold | 1.47 × 10^-05^ | 198–239 | pfam01367 |
|  |  |  |  |  | H3TH domain of the 5′–3′ exonuclease H3TH_53EXO  Taq DNA polymerase I and homologs | 4.13 × 10^-05^ | 198–235 | cd09898 |
|  |  |  |  |  | DNA polymerase I; Provisional | 9.35 × 10^-06^ | 132–239 | PRK05755 |
|  |  |  |  |  | 5′–3′ exonuclease | 1.18 × 10^-05^ | 132–239 | smart0047 |
|  |  |  |  |  | DNA polymerase I | 2.28 × 10^-05^ | 84–236 | TIGR00593 |
| ORF28 | 15552 | 16331 | + | 259 | RNase H superfamily | 3.56 × 10^-09^ | 8–186 | pfam13482 |
|  |  |  |  |  | EXOIII exonuclease domain | 6.83 × 10^-05^ | 72–137 | smart00479 |
|  |  |  |  |  | InsA transposase | 5.22 × 10^-05^ | 198–238 | COG3677 |
| ORF31 | 17015 | 17860 | + | 281 | adenylation domain | 1.77 × 10^-10^ | 21–195 | cd06846 |
|  |  |  |  |  | DNA ligase OB-like domain | 1.22 × 10^-07^ | 222–273 | pfam14743 |
|  |  |  |  |  | ATP dependent DNA ligase domain | 1.77 × 10^-07^ | 33–195 | pfam01068 |
|  |  |  |  |  | oligonucleotide/oligosaccharide binding (OB)-fold domain | 3.06 × 10^-07^ | 221–273 | cd08041 |
|  |  |  |  |  | DNA ligase; Provisional | 1.41 × 10^-11^ | 21–276 | PHA02587 |
|  |  |  |  |  | DNA ligase D, ligase domain | 9.36 × 10^-06^ | 85–276 | TIGR02779 |
| ORF32 | 17869 | 20283 | + | 804 | DNA-dependent RNA polymerase | 1.48 × 10^-132^ | 420–803 | pfam00940 |
|  |  |  |  |  | DNA-directed RNA polymerase N-terminal | 1.93 × 10^-05^ | 4–285 | pfam14700 |
|  |  |  |  |  | mitochondrial DNA-directed RNA polymerase | 1.16 × 10^-127^ | 4–804 | COG5108 |
| ORF35 | 21404 | 22930 | + | 508 | bacteriophage head to tail connecting protein | 1.29 × 10^-51^ | 13–468 | pfam12236 |
| ORF37 | 23820 | 24815 | + | 331 | capsid protein | 4.53 × 10^-18^ | 25–328 | PHA02004 |
| ORF38 | 24897 | 25502 | + | 201 | tail tubular protein A | 9.16 × 10^-40^ | 4–181 | PHA00428 |
| ORF42 | 31250 | 35209 | + | 1319 | lytic transglycosylase (LT) and goose egg white lysozyme (GEWL) domain. | 1.05 × 10^-13^ | 27–127 | cd00254 |
|  |  |  |  |  | putative internal virion protein; provisional | 3.07 × 10^-12^ | 723–1245 | PHA03415 |
|  |  |  |  |  | transglycosylase SLT domain | 4.13 × 10^-05^ | 32–109 | pfam01464 |
|  |  |  |  |  | internal virion protein D | 1.91 × 10^-11^ | 7-1292 | PHA00368 |
| ORF43 | 35267 | 37195 | + | 642 | phage T7 tail fiber protein | 1.28 × 10^-11^ | 6–135 | pfam03906 |
| ORF44 | 37195 | 37605 | + | 136 | phage tail assembly chaperone protein | 1.15 × 10^-15^ | 71–133 | pfam16778 |
